# 3-{[5-(4-Bromo­phen­yl)imidazo[2,1-*b*][1,3,4]thia­diazol-2-yl]meth­yl}-1,2-benzoxazole

**DOI:** 10.1107/S1600536810052232

**Published:** 2010-12-18

**Authors:** Afshan Banu, Mohamed Ziaulla, Noor Shahina Begum, Ravi S. Lamani, I. M. Khazi

**Affiliations:** a Department of Studies in Chemistry, Bangalore University, Bangalore 560 001, India; b Department of Chemistry, Karnatak University, Dharwad 580 003, India

## Abstract

In the title compound, C_18_H_11_BrN_4_OS, the imidazothia­diazole and benzisoxazole rings are individually planar with maximum deviations of 0.025 (3) 0.015 (4) Å, respectively, and are inclined at an angle of 23.51 (7)° with respect to each other. The planes of the imidazothia­diazole and bromo­phenyl rings are inclined at an angle of 27.34 (3)°. In the crystal, inter­molecular C—H⋯N inter­actions result in chains of mol­ecules along the *b* and *c* axes. Moreover, C—H⋯O inter­actions result in centrosymmetric head-to-head dimers with *R*_2_^2^(24) graph-set motifs. The mol­ecular packing is further stabilized by π–π stacking inter­actions between the imidazole rings with a shortest centroid–centroid distance of 3.492 (3) Å. In addition, C—H⋯π inter­actions are observed in the crystal structure.

## Related literature

For the biological activity of benzisoxazole derivatives, see: Priya *et al.* (2005 ▶). For the preparation of the title compound, see: Lamani *et al.* (2009 ▶). For a related structure, see: Sun & Zhang (2009 ▶). For graph-set notation, see: Bernstein *et al.* (1995 ▶).
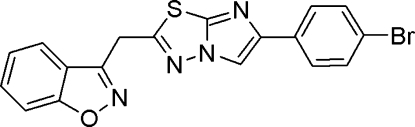


## Experimental

### 

#### Crystal data

C_18_H_11_BrN_4_OS
*M* *_r_* = 411.28
Monoclinic, 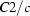

*a* = 38.985 (17) Å
*b* = 5.764 (3) Å
*c* = 14.925 (6) Å
β = 109.191 (13)°
*V* = 3167 (2) Å^3^
*Z* = 8
Mo *K*α radiation
μ = 2.74 mm^−1^
*T* = 423 K
0.18 × 0.16 × 0.16 mm

#### Data collection

Bruker SMART APEX CCD detector diffractometer
Absorption correction: multi-scan (*SADABS*; Bruker, 1998 ▶) *T*_min_ = 0.638, *T*_max_ = 0.668
8879 measured reflections
3432 independent reflections
2534 reflections with *I* > 2σ(*I*)
*R*_int_ = 0.081

#### Refinement

*R*[*F* ^2^ > 2σ(*F* ^2^)] = 0.052
*wR*(*F* ^2^) = 0.142
*S* = 1.02
3432 reflections
226 parameters
H-atom parameters constrained
Δρ_max_ = 1.10 e Å^−3^
Δρ_min_ = −1.02 e Å^−3^

### 

Data collection: *SMART* (Bruker, 1998 ▶); cell refinement: *SAINT-Plus* (Bruker, 1998 ▶); data reduction: *SAINT-Plus*; program(s) used to solve structure: *SHELXS97* (Sheldrick, 2008 ▶); program(s) used to refine structure: *SHELXL97* (Sheldrick, 2008 ▶); molecular graphics: *ORTEP-3* (Farrugia, 1997 ▶) and *CAMERON* (Watkin *et al.*, 1996) ▶; software used to prepare material for publication: *WinGX* (Farrugia, 1999 ▶).

## Supplementary Material

Crystal structure: contains datablocks global, I. DOI: 10.1107/S1600536810052232/pv2359sup1.cif

Structure factors: contains datablocks I. DOI: 10.1107/S1600536810052232/pv2359Isup2.hkl

Additional supplementary materials:  crystallographic information; 3D view; checkCIF report

## Figures and Tables

**Table 1:** Hydrogen-bond geometry (Å, °) *Cg*4 and *Cg*5 are the centroids of the C1–C6 and C13–C18 rings, respectively.

*D*—H⋯*A*	*D*—H	H⋯*A*	*D*⋯*A*	*D*—H⋯*A*
C2—H2⋯O1^i^	0.93	2.38	3.219 (6)	150
C8—H8⋯N1^ii^	0.93	2.60	3.469 (6)	156
C11—H11*B*⋯N1^iii^	0.97	2.48	3.358 (6)	150
C4—H4⋯*Cg*5^iii^	0.93	2.96	3.554 (5)	123
C18—H18⋯*Cg*4^iii^	0.93	2.83	3.496 (5)	130

Symmetry codes: (i) 
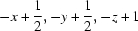
; (ii) 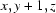; (iii) 
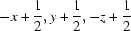
.

**Fig. 1.**
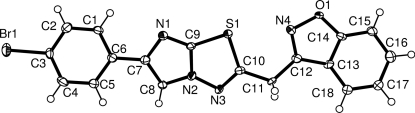
ORTEP (Farrugia, 1997) view of the title compound, showing 50% probability ellipsoids and the atom numbering scheme.

**Fig. 2.**
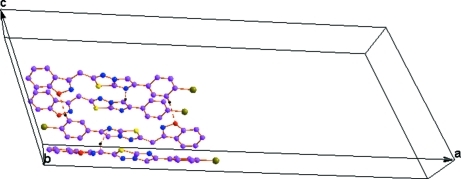
A unit cell packing of the title compound showing intermolecular interactions with dotted lines. H-atoms not involved in hydrogen bonding have been excluded.

## supplementary crystallographic information

### Comment

Benzisoxazole derivatives are known to possess important biological activities (Priya *et al.*, 2005). In view of increasing importance of the heterocycles in pharmaceutical and biological fields, it was considered of interest to synthesize some new chemical entities incorporating two active pharmacophores in a single molecular frame work and to evaluate their biological activities. In the title compound (Fig. 1), the fused imidazothiadiazole ring system is linked to a benzisoxazole and a bromophenyl moieties. The imidazothiadiazole (S1/N1–N3/C7–C10) and benzisoxazole (O1/N4/C12–C18) rings are individually planar similar to those reported earlier (Sun & Zhang, 2009) with maximum deviations 0.025 (3) Å for N2 and 0.015 (4) Å for C17, respectively. The mean-plane of benzisoxazole makes a dihedral angle of 23.51 (7)° with the mean-plane of the imidazothiadiazole ring. The planes of the imidazothiadiazole and bromophenyl rings are inclined at an angle 27.34 (3)° with each other. The differences in bond lengths S1—C9 (1.734 (5) Å) and S1—C10 (1.757 (5) Å) indicate that the resonance effect caused by the imidazole ring is stronger than that caused by the thiadiazole ring. In the crystal structure, intermolecular interactions C8—H8···N1 result in chains of molecules along the *c*-axis and C11—H11B···N1 interactions result in chains of molecules along the *b*-axis. Moreover, C2—H2···O1 interactions result in centrosymmetric head-to-head dimers corresponding to *R*_2_^2^(24) graph set motif (Bernstein *et al.*, 1995) (Fig. 2). The molecular packing is further stabilized by π-π stacking interactions between imidazo rings (*Cg*3) with the shortest centroid–centroid distance 3.492 (3) Å. In addition, π-ring interactions of the type C—H···*Cg* (*Cg* being the centroids of rings C1–C6 and C13–C18) are also observed in the crystal structure; details have been provided in Table 1.

### Experimental

The title compound was synthesized by following the procedure reported earlier (Lamani *et al.*, 2009) and suitable crystals for X-ray crystallographic analysis were grown from a solution of dimethylformamide by slow evaporation at room temperature.

### Refinement

The H atoms were placed at calculated positions in the riding model approximation with N—H = 0.86 and C—H = 0.97 Å, and *U*_iso_(H) = 1.2*U*_eq_(N/C).

### Crystal data

**Table:**

C_18_H_11_BrN_4_OS	*F*(000) = 1648
*M**_r_* = 411.28	*D*_x_ = 1.725 Mg m^−^^3^
Monoclinic, *C*2/*c*	Mo *K*α radiation, λ = 0.71073 Å
Hall symbol: -C 2yc	Cell parameters from 3432 reflections
*a* = 38.985 (17) Å	θ = 2.2–27.0°
*b* = 5.764 (3) Å	µ = 2.74 mm^−^^1^
*c* = 14.925 (6) Å	*T* = 423 K
β = 109.191 (13)°	Block, yellow
*V* = 3167 (2) Å^3^	0.18 × 0.16 × 0.16 mm
*Z* = 8	

### Data collection

**Table:**

Bruker SMART APEX CCD detector diffractometer	3432 independent reflections
Radiation source: Enhance (Mo) X-ray Source	2534 reflections with *I* > 2σ(*I*)
graphite	*R*_int_ = 0.081
ω scans	θ_max_ = 27.0°, θ_min_ = 2.2°
Absorption correction: multi-scan (*SADABS*; Bruker, 1998)	*h* = −44→49
*T*_min_ = 0.638, *T*_max_ = 0.668	*k* = −7→7
8879 measured reflections	*l* = −16→19

### Refinement

**Table:**

Refinement on *F*^2^	Primary atom site location: structure-invariant direct methods
Least-squares matrix: full	Secondary atom site location: difference Fourier map
*R*[*F*^2^ > 2σ(*F*^2^)] = 0.052	Hydrogen site location: inferred from neighbouring sites
*wR*(*F*^2^) = 0.142	H-atom parameters constrained
*S* = 1.02	*w* = 1/[σ^2^(*F*_o_^2^) + (0.0787*P*)^2^] where *P* = (*F*_o_^2^ + 2*F*_c_^2^)/3
3432 reflections	(Δ/σ)_max_ < 0.001
226 parameters	Δρ_max_ = 1.10 e Å^−^^3^
0 restraints	Δρ_min_ = −1.02 e Å^−^^3^

### Special details

**Table:**

Experimental. The compound was synthesized by following the procedure given in Lamani *et al.*, (2009)
Geometry. All s.u.'s (except the s.u. in the dihedral angle between two l.s. planes) are estimated using the full covariance matrix. The cell s.u.'s are taken into account individually in the estimation of s.u.'s in distances, angles and torsion angles; correlations between s.u.'s in cell parameters are only used when they are defined by crystal symmetry. An approximate (isotropic) treatment of cell s.u.'s is used for estimating s.u.'s involving l.s. planes.
Refinement. Refinement of *F*^2^ against ALL reflections. The weighted *R*-factor *wR* and goodness of fit *S* are based on *F*^2^, conventional *R*-factors *R* are based on *F*, with *F* set to zero for negative *F*^2^. The threshold expression of *F*^2^ > σ(*F*^2^) is used only for calculating *R*-factors(gt) *etc*. and is not relevant to the choice of reflections for refinement. *R*-factors based on *F*^2^ are statistically about twice as large as those based on *F*, and *R*- factors based on ALL data will be even larger.

### Fractional atomic coordinates and isotropic or equivalent isotropic displacement parameters (Å^2^)

**Table:**

	*x*	*y*	*z*	*U*_iso_*/*U*_eq_	
C1	0.14779 (11)	0.5054 (7)	0.4239 (3)	0.0178 (8)	
H1	0.1636	0.3813	0.4453	0.021*	
C2	0.11246 (11)	0.4910 (8)	0.4268 (3)	0.0213 (9)	
H2	0.1048	0.3593	0.4507	0.026*	
C3	0.08923 (11)	0.6744 (7)	0.3938 (3)	0.0193 (9)	
C4	0.10031 (12)	0.8734 (7)	0.3593 (3)	0.0222 (9)	
H4	0.0844	0.9972	0.3382	0.027*	
C5	0.13517 (11)	0.8857 (7)	0.3567 (3)	0.0200 (9)	
H5	0.1426	1.0183	0.3327	0.024*	
C6	0.15970 (11)	0.7034 (7)	0.3892 (3)	0.0163 (8)	
C7	0.19675 (11)	0.7159 (7)	0.3855 (3)	0.0165 (8)	
C8	0.21618 (11)	0.9130 (7)	0.3817 (3)	0.0175 (8)	
H8	0.2091	1.0667	0.3831	0.021*	
C9	0.24700 (11)	0.5928 (7)	0.3759 (3)	0.0166 (8)	
C10	0.30080 (11)	0.7731 (7)	0.3592 (3)	0.0178 (8)	
C11	0.33397 (10)	0.8402 (7)	0.3361 (3)	0.0168 (8)	
H11A	0.3421	0.9897	0.3651	0.020*	
H11B	0.3269	0.8616	0.2679	0.020*	
C12	0.36595 (11)	0.6779 (7)	0.3653 (3)	0.0178 (8)	
C13	0.40073 (11)	0.7153 (7)	0.3545 (3)	0.0162 (8)	
C14	0.41996 (11)	0.5140 (7)	0.3897 (3)	0.0187 (9)	
C15	0.45543 (11)	0.4746 (8)	0.3946 (3)	0.0212 (9)	
H15	0.4677	0.3387	0.4197	0.025*	
C16	0.47149 (12)	0.6516 (8)	0.3594 (3)	0.0236 (9)	
H16	0.4953	0.6341	0.3602	0.028*	
C17	0.45273 (11)	0.8557 (8)	0.3228 (3)	0.0216 (9)	
H17	0.4643	0.9701	0.2994	0.026*	
C18	0.41745 (12)	0.8920 (8)	0.3207 (3)	0.0207 (9)	
H18	0.4053	1.0295	0.2974	0.025*	
Br1	0.040967 (11)	0.65163 (8)	0.39690 (3)	0.02724 (18)	
N1	0.21625 (9)	0.5149 (6)	0.3825 (2)	0.0171 (7)	
N2	0.24852 (9)	0.8310 (6)	0.3753 (2)	0.0172 (7)	
N3	0.27848 (9)	0.9330 (6)	0.3650 (2)	0.0198 (7)	
N4	0.36389 (9)	0.4744 (6)	0.4012 (2)	0.0210 (8)	
O1	0.39822 (8)	0.3642 (5)	0.4181 (2)	0.0221 (7)	
S1	0.28738 (3)	0.48552 (18)	0.36718 (7)	0.0188 (2)	

### Atomic displacement parameters (Å^2^)

**Table:**

	*U*^11^	*U*^22^	*U*^33^	*U*^12^	*U*^13^	*U*^23^
C1	0.0170 (19)	0.009 (2)	0.027 (2)	0.0004 (16)	0.0056 (16)	0.0014 (15)
C2	0.022 (2)	0.019 (2)	0.024 (2)	−0.0042 (18)	0.0089 (17)	0.0014 (17)
C3	0.0144 (19)	0.022 (2)	0.0217 (19)	−0.0019 (17)	0.0057 (16)	−0.0048 (16)
C4	0.022 (2)	0.016 (2)	0.027 (2)	0.0043 (18)	0.0051 (18)	−0.0010 (16)
C5	0.020 (2)	0.015 (2)	0.027 (2)	−0.0028 (17)	0.0088 (17)	−0.0008 (16)
C6	0.0138 (19)	0.015 (2)	0.0197 (19)	0.0023 (16)	0.0050 (15)	−0.0014 (15)
C7	0.0150 (19)	0.015 (2)	0.0200 (19)	−0.0008 (16)	0.0056 (16)	0.0012 (15)
C8	0.0155 (19)	0.013 (2)	0.025 (2)	0.0029 (16)	0.0085 (17)	−0.0007 (15)
C9	0.017 (2)	0.012 (2)	0.0201 (19)	−0.0007 (16)	0.0043 (16)	0.0005 (14)
C10	0.016 (2)	0.013 (2)	0.022 (2)	−0.0030 (17)	0.0033 (16)	0.0018 (16)
C11	0.0127 (19)	0.014 (2)	0.024 (2)	−0.0023 (16)	0.0063 (16)	0.0041 (16)
C12	0.017 (2)	0.011 (2)	0.0223 (19)	−0.0011 (16)	0.0028 (16)	−0.0012 (15)
C13	0.016 (2)	0.011 (2)	0.0196 (19)	0.0012 (16)	0.0028 (16)	0.0006 (14)
C14	0.018 (2)	0.012 (2)	0.026 (2)	0.0009 (16)	0.0072 (16)	0.0017 (16)
C15	0.017 (2)	0.014 (2)	0.030 (2)	0.0055 (17)	0.0052 (17)	−0.0019 (17)
C16	0.018 (2)	0.025 (3)	0.027 (2)	−0.0016 (19)	0.0060 (17)	−0.0013 (18)
C17	0.019 (2)	0.020 (2)	0.027 (2)	−0.0024 (18)	0.0091 (17)	0.0020 (17)
C18	0.021 (2)	0.016 (2)	0.024 (2)	−0.0013 (17)	0.0063 (17)	0.0019 (16)
Br1	0.0161 (2)	0.0308 (3)	0.0357 (3)	−0.00248 (19)	0.00975 (18)	−0.00722 (19)
N1	0.0158 (16)	0.0112 (18)	0.0244 (17)	0.0023 (14)	0.0066 (14)	−0.0005 (13)
N2	0.0159 (17)	0.0112 (18)	0.0254 (17)	−0.0007 (14)	0.0082 (14)	−0.0012 (13)
N3	0.0128 (16)	0.0151 (19)	0.0324 (19)	−0.0055 (14)	0.0089 (15)	−0.0014 (15)
N4	0.0151 (17)	0.018 (2)	0.0303 (19)	0.0007 (15)	0.0085 (14)	0.0026 (14)
O1	0.0177 (15)	0.0107 (15)	0.0403 (17)	0.0047 (12)	0.0127 (13)	0.0085 (12)
S1	0.0156 (5)	0.0106 (5)	0.0308 (5)	−0.0009 (4)	0.0085 (4)	−0.0002 (4)

### Geometric parameters (Å, °)

**Table:**

C1—C6	1.393 (6)	C10—C11	1.495 (5)
C1—C2	1.395 (6)	C10—S1	1.754 (4)
C1—H1	0.9300	C11—C12	1.504 (5)
C2—C3	1.374 (6)	C11—H11A	0.9700
C2—H2	0.9300	C11—H11B	0.9700
C3—C4	1.383 (6)	C12—N4	1.303 (5)
C3—Br1	1.902 (4)	C12—C13	1.433 (6)
C4—C5	1.374 (6)	C13—C14	1.387 (5)
C4—H4	0.9300	C13—C18	1.390 (6)
C5—C6	1.396 (6)	C14—O1	1.371 (5)
C5—H5	0.9300	C14—C15	1.379 (6)
C6—C7	1.465 (5)	C15—C16	1.387 (6)
C7—C8	1.377 (6)	C15—H15	0.9300
C7—N1	1.395 (5)	C16—C17	1.398 (6)
C8—N2	1.379 (5)	C16—H16	0.9300
C8—H8	0.9300	C17—C18	1.381 (6)
C9—N1	1.314 (5)	C17—H17	0.9300
C9—N2	1.374 (5)	C18—H18	0.9300
C9—S1	1.736 (4)	N2—N3	1.360 (5)
C10—N3	1.290 (5)	N4—O1	1.427 (4)
			
C6—C1—C2	120.9 (4)	C10—C11—H11B	107.8
C6—C1—H1	119.6	C12—C11—H11B	107.8
C2—C1—H1	119.6	H11A—C11—H11B	107.1
C3—C2—C1	119.0 (4)	N4—C12—C13	111.7 (4)
C3—C2—H2	120.5	N4—C12—C11	121.5 (4)
C1—C2—H2	120.5	C13—C12—C11	126.7 (4)
C2—C3—C4	121.5 (4)	C14—C13—C18	119.4 (4)
C2—C3—Br1	118.6 (3)	C14—C13—C12	104.2 (3)
C4—C3—Br1	119.9 (3)	C18—C13—C12	136.4 (4)
C5—C4—C3	119.1 (4)	O1—C14—C15	126.2 (4)
C5—C4—H4	120.5	O1—C14—C13	109.4 (3)
C3—C4—H4	120.5	C15—C14—C13	124.4 (4)
C4—C5—C6	121.5 (4)	C14—C15—C16	115.4 (4)
C4—C5—H5	119.3	C14—C15—H15	122.3
C6—C5—H5	119.3	C16—C15—H15	122.3
C1—C6—C5	118.1 (4)	C15—C16—C17	121.6 (4)
C1—C6—C7	120.5 (4)	C15—C16—H16	119.2
C5—C6—C7	121.4 (4)	C17—C16—H16	119.2
C8—C7—N1	111.8 (4)	C18—C17—C16	121.7 (4)
C8—C7—C6	127.2 (4)	C18—C17—H17	119.2
N1—C7—C6	121.0 (4)	C16—C17—H17	119.2
C7—C8—N2	104.4 (4)	C17—C18—C13	117.6 (4)
C7—C8—H8	127.8	C17—C18—H18	121.2
N2—C8—H8	127.8	C13—C18—H18	121.2
N1—C9—N2	112.6 (4)	C9—N1—C7	103.8 (3)
N1—C9—S1	139.1 (3)	N3—N2—C9	118.2 (3)
N2—C9—S1	108.3 (3)	N3—N2—C8	134.3 (4)
N3—C10—C11	119.0 (4)	C9—N2—C8	107.4 (3)
N3—C10—S1	116.6 (3)	C10—N3—N2	108.8 (3)
C11—C10—S1	124.1 (3)	C12—N4—O1	107.0 (3)
C10—C11—C12	118.0 (3)	C14—O1—N4	107.7 (3)
C10—C11—H11A	107.8	C9—S1—C10	88.1 (2)
C12—C11—H11A	107.8		
			
C6—C1—C2—C3	0.8 (6)	C13—C14—C15—C16	−1.3 (6)
C1—C2—C3—C4	−1.0 (6)	C14—C15—C16—C17	0.7 (6)
C1—C2—C3—Br1	179.3 (3)	C15—C16—C17—C18	0.6 (6)
C2—C3—C4—C5	1.1 (6)	C16—C17—C18—C13	−1.3 (6)
Br1—C3—C4—C5	−179.3 (3)	C14—C13—C18—C17	0.8 (6)
C3—C4—C5—C6	−0.9 (6)	C12—C13—C18—C17	−179.8 (4)
C2—C1—C6—C5	−0.7 (6)	N2—C9—N1—C7	0.5 (4)
C2—C1—C6—C7	−179.4 (3)	S1—C9—N1—C7	−179.3 (4)
C4—C5—C6—C1	0.7 (6)	C8—C7—N1—C9	−0.6 (4)
C4—C5—C6—C7	179.4 (4)	C6—C7—N1—C9	177.6 (3)
C1—C6—C7—C8	−158.6 (4)	N1—C9—N2—N3	−177.5 (3)
C5—C6—C7—C8	22.8 (6)	S1—C9—N2—N3	2.4 (4)
C1—C6—C7—N1	23.5 (5)	N1—C9—N2—C8	−0.2 (4)
C5—C6—C7—N1	−155.2 (4)	S1—C9—N2—C8	179.7 (3)
N1—C7—C8—N2	0.5 (4)	C7—C8—N2—N3	176.5 (4)
C6—C7—C8—N2	−177.6 (4)	C7—C8—N2—C9	−0.2 (4)
N3—C10—C11—C12	156.9 (4)	C11—C10—N3—N2	172.8 (3)
S1—C10—C11—C12	−30.0 (5)	S1—C10—N3—N2	−0.8 (4)
C10—C11—C12—N4	6.7 (6)	C9—N2—N3—C10	−1.1 (5)
C10—C11—C12—C13	−176.4 (4)	C8—N2—N3—C10	−177.5 (4)
N4—C12—C13—C14	−1.2 (4)	C13—C12—N4—O1	0.9 (4)
C11—C12—C13—C14	−178.4 (4)	C11—C12—N4—O1	178.3 (3)
N4—C12—C13—C18	179.3 (4)	C15—C14—O1—N4	179.5 (4)
C11—C12—C13—C18	2.2 (7)	C13—C14—O1—N4	−0.5 (4)
C18—C13—C14—O1	−179.4 (3)	C12—N4—O1—C14	−0.3 (4)
C12—C13—C14—O1	1.0 (4)	N1—C9—S1—C10	177.7 (5)
C18—C13—C14—C15	0.5 (6)	N2—C9—S1—C10	−2.1 (3)
C12—C13—C14—C15	−179.0 (4)	N3—C10—S1—C9	1.8 (3)
O1—C14—C15—C16	178.7 (4)	C11—C10—S1—C9	−171.5 (3)

### Hydrogen-bond geometry (Å, °)

**Table:**

Cg4 and Cg5 are the centroids of the C1–C6 and C13–C18 rings, respectively.

**Table:**

*D*—H···*A*	*D*—H	H···*A*	*D*···*A*	*D*—H···*A*
C2—H2···O1^i^	0.93	2.38	3.219 (6)	150
C8—H8···N1^ii^	0.93	2.60	3.469 (6)	156
C11—H11B···N1^iii^	0.97	2.48	3.358 (6)	150
C4—H4···Cg5^iii^	0.93	2.96	3.554 (5)	123
C18—H18···Cg4^iii^	0.93	2.83	3.496 (5)	130

Symmetry codes: (i) −*x*+1/2, −*y*+1/2, −*z*+1; (ii) *x*, *y*+1, *z*; (iii) −*x*+1/2, *y*+1/2, −*z*+1/2.
